# Augmented reality for supporting the interaction between pedestrians and automated vehicles: an experimental outdoor study

**DOI:** 10.3389/frobt.2024.1324060

**Published:** 2024-01-30

**Authors:** Thomas K. Aleva, Wilbert Tabone, Dimitra Dodou, Joost C. F. de Winter

**Affiliations:** Faculty of Mechanical Engineering, Delft University of Technology, Delft, Netherlands

**Keywords:** augmented reality, pedestrian safety, anchoring, see-through AR, head-mounted device (HMD)

## Abstract

**Introduction:** Communication from automated vehicles (AVs) to pedestrians using augmented reality (AR) could positively contribute to traffic safety. However, previous AR research for pedestrians was mainly conducted through online questionnaires or experiments in virtual environments instead of real ones.

**Methods:** In this study, 28 participants conducted trials outdoors with an approaching AV and were supported by four different AR interfaces. The AR experience was created by having participants wear a Varjo XR-3 headset with see-through functionality, with the AV and AR elements virtually overlaid onto the real environment. The AR interfaces were vehicle-locked (*Planes on vehicle*), world-locked (*Fixed pedestrian lights*, *Virtual fence*), or head-locked (*Pedestrian lights HUD*). Participants had to hold down a button when they felt it was safe to cross, and their opinions were obtained through rating scales, interviews, and a questionnaire.

**Results:** The results showed that participants had a subjective preference for AR interfaces over no AR interface. Furthermore, the *Pedestrian lights HUD* was more effective than no AR interface in a statistically significant manner, as it led to participants more frequently keeping the button pressed. The *Fixed pedestrian lights* scored lower than the other interfaces, presumably due to low saliency and the fact that participants had to visually identify both this AR interface and the AV.

**Discussion:** In conclusion, while users favour AR in AV-pedestrian interactions over no AR, its effectiveness depends on design factors like location, visibility, and visual attention demands. In conclusion, this work provides important insights into the use of AR outdoors. The findings illustrate that, in these circumstances, a clear and easily interpretable AR interface is of key importance.

## Introduction

Every year, 300,000 pedestrian deaths occur worldwide, accounting for 23% of all road fatalities (World Health Organization, 2018). The United Nations has set a target within its Sustainable Development Goals to halve the number of traffic deaths by 2030 (United Nations, 2020). In this resolution (A/RES/74/299), it was also noted that “continuous progress of automotive and digital technologies could improve road safety, including through the progressive development of highly and fully automated vehicles in road traffic” (United Nations, 2020, p. 4).

One possibility to improve the safety of pedestrians is to equip automated vehicles (AVs) with better sensors and intelligence so that they can respond earlier, in order to reduce collisions with vulnerable road users (Jungmann et al., 2020; Paiva et al., 2021). Another possibility is the introduction of AV-to-pedestrian communication technologies. Previous research has shown that with displays on the outside of the AV, called external human-machine interfaces or eHMIs, pedestrians can make more effective road-crossing decisions (Dey et al., 2020; Bazilinskyy et al., 2021; Bindschädel et al., 2022). However, eHMIs have certain disadvantages in that they typically cannot address an individual pedestrian (Colley et al., 2020; Tran et al., 2023) and that they can be difficult to perceive in some cases, for example, due to an occlusion by another object (Troel-Madec et al., 2019; Dey et al., 2022).

Recently, AV-to-pedestrian communication via wearable devices has been proposed (Hasan and Hasan, 2022; Gelbal et al., 2023; Lakhdhir et al., 2023). More specifically, augmented reality (AR), defined as a technology that overlays virtual information onto the real world, appears to be a promising solution for communicating with pedestrians (Tabone et al., 2021a). On the one hand, the use of AR might sound unusual and undesirable; after all, it may be questioned whether pedestrians would want to rely on an expensive headset in order to move safely through traffic (e.g., Tabone et al., 2021a; Berge et al., 2022). At the same time, given that other AR technologies such as car head-up displays (HUDs) (Transparency Market Research, 2023) and Google Maps visual overlays on mobile phones (Google, 2020), once seen as futuristic, are now common, it is conceivable that in the future, pedestrians will receive visual assistance through AR glasses.

Although AR is a potentially promising technology for pedestrians, still little research exists on this topic. One of the problems is that AR is currently challenging to implement, with only the most recent headsets capable of creating a compelling and user-friendly experience (e.g., Microsoft Hololens 2, Varjo XR 3, Magic Leap 2). While exceptions exist (e.g., Kang et al., 2023; Zhang et al., 2023), much previous AR research for pedestrians has limitations: some studies focus solely on the orienting phase without any form of human-subject evaluation (Tabone et al., 2021b; Tong et al., 2021), others use questionnaires with photos or videos (Hesenius et al., 2018; Tabone et al., 2023a; Wilbrink et al., 2023), and still others test AR concepts in immersive virtual reality (VR) environments (Pratticò et al., 2021; Tran et al., 2022; Tabone et al., 2023b; Malik et al., 2023). A critical note that should be made in this last point is that, although valid results can be obtained in VR, this is strictly speaking not AR. After all, merely adding virtual displays within a completely virtual environment is still considered VR, rather than AR. AR is different from VR in that AR incorporates elements of the physical world into the user’s experience, creating a blend of virtual and real-world elements (Rauschnabel et al., 2022).

In this study, we share the findings from an outdoor experiment involving participants using an AR headset. In addition to the AR interfaces, the approaching AV was also simulated. The use of a virtual AV, as opposed to a real one, ensured consistent experimental conditions and simplified the process of attaching AR elements to the moving AV. Our approach serves as a stepping stone to a future full AR experience, in which pedestrians can walk around untethered while the AR headset wirelessly communicates with an actual AV. Our use of AR, where virtual road users coexist with real humans in an outdoor space, has been applied by several others before, though not in the context of AV-pedestrian communication (Maruhn et al., 2020; Kamalasanan et al., 2022). Our methodology also corresponds with other realistic simulation methods, such as displaying virtual road users in a real car (Bokc et al., 2007; Hussain et al., 2013; Sheridan, 2016; Butenuth et al., 2017; Feng et al., 2018), a technique that supersedes driving simulators which inherently offer limited visual and motion cues. In our approach, while the environment remains the real world, it is enhanced with simulated experimental stimuli.

In the current experiment, four different AR interfaces and a no-AR-interface baseline condition were compared among human participants. The focus was on whether these AR interfaces made participants feel safe to cross, as well as subjective qualities including whether the AR interface was found to be intuitive and was accepted. The interfaces used in this study were adopted from three prior works: an AR concept design study (Tabone et al., 2021b), an online questionnaire with video clips in which participants provided subjective ratings (Tabone et al., 2023a), and a CAVE-based simulator study in which participants crossed a virtual road (Tabone et al., 2023b). Our goal was to investigate whether the results we found in the current experiment correspond with the earlier research among human participants (Tabone et al., 2023a; 2023b).

The tested AR interfaces differed in terms of their anchoring techniques: the AR interface was either vehicle-locked, world-locked, or head-locked, three methods that bring fundamentally different demands regarding the user’s visual attention (Lebeck et al., 2017; Lingam et al., 2023; Peereboom et al., 2023; Tabone et al., 2023b). A vehicle-locked AR interface entails, just like an eHMI, that implicit communication in the form of the speed of and distance to the AV and explicit AR cues are congruent in time and place. A head-locked AR interface, on the other hand, is always visible but requires that the AV is looked at to confirm the cues of the AR interface. Finally, a world-locked interface requires attention to be distributed between the AV and the AR interface, with the possible advantage that the AR interface can be presented at a fixed and familiar location.

For illustration, Lingam et al. (2023) conducted a study using a VR driving simulator to investigate whether eHMIs were better positioned on the roof of an approaching AV, or integrated into the road infrastructure, resembling a traffic light configuration. Their results showed that both solutions were more favoured by the car drivers than the absence of any signalling. However, each concept presented its own set of advantages and disadvantages. The advantage of the infrastructure solution (i.e., world-locked signal) was that the signals were positioned at a fixed and accessible location before the intersection, and the driver could see this from a distance. An eHMI on the AV (i.e., vehicle-locked signal), on the other hand, required that the driver first turn their head/eyes to see the AV with its eHMI; once identified, however, the driver could directly infer what the AV was going to do, as deduced from its speed and the eHMI signal.

The current study, conducted with pedestrians outdoors instead of in VR, aimed to determine whether similar strengths and weaknesses could be identified for various AR anchoring methods. We implement four AR interface concepts, taken from Tabone et al. (2023b): 1) *Virtual fence*, a world-locked interface that is highly visible, yet may impart a false sense of security and which partially occludes the environment due to its semi-transparent walls, 2) *Fixed pedestrian lights*, another world-locked interface; it has a familiar design and is smaller in stature compared to the *Virtual fence*, 3) *Pedestrian lights HUD*, a head-locked interface similar to a conventional traffic light, but always visible in the users field of view, and 4) *Planes on vehicle*, a vehicle-locked AR interface similar to an eHMI. We adopted the designs from Tabone et al. (2023a, 2023b) without design improvements. Note that several insurmountable differences exist between the AR implementation of this study and that of the online and VR studies by Tabone et al. (2023a, 2023b), such as in timing and visibility in the external environment, thereby making an exact numerical comparison not our objective. Instead, we opted for a qualitative comparison, focusing on the effects of the anchoring method. In addition to comparing different AR interfaces with no AR interface, one of the objectives of this research was to document, accompanied by Unity source code, how AR research in the outdoor environment can be conducted.

## Methods

### Participants

Participants were recruited through personal networks, without the offer of financial reimbursement or other incentives. The study included a total of 28 persons, comprising 23 males and 5 females, with ages ranging from 19 to 59 years. The average age was 27.2 years, with a standard deviation of 9.2 years. In response to the intake questionnaire item ‘Do you use any seeing aids?’, 19 participants answered ‘no’, 5 answered ‘yes, glasses’, and 4 answered ‘yes, contact lenses’. We did not record whether participants wore glasses or contact lenses during the experiment. However, it was observed that at least one participant wore their glasses under the headset, without noticeable problems.

The intake questionnaire further indicated that 19 of the participants were students. Twenty-six participants indicated being Dutch, one Chinese, and the nationality of one participant was unknown. Twenty-six participants held a driver’s licence, for an average of 8.0 (*SD* = 7.3) years. A total of 17 participants had used a VR headset before while 9 participants had used an AR headset before. Daily walking time was less than 15 min for 7 participants, 15–30 min for 12 participants, and more than 30 min for 9 participants. Cycling was the primary mode of transportation for most participants (22 out of 28). A test for colour blindness showed that one participant was colorblind. This person was not excluded from participation, since the AR interfaces featured redundancy gain in that they did not solely rely on red/green colours, but also used icons, movement, or stimulus location cues. Additionally, the inclusion of this participant was considered beneficial for gaining valuable insights.

Each participant provided written informed consent. The experiment procedure was approved by the TU Delft Human Research Ethics Committee, approval no. 3054.

### Materials and settings

The experiment took place in a designated area of the Delft University of Technology campus, which was closed to normal traffic. It was conducted on an Alienware PC powered by an Intel^®^ Core™i7-9700K CPU at 3.60 GHz, equipped with 16 GB RAM and a Nvidia GeForce RTX 2080 Ti GPU. The AR software ran in Unity 2021.3.13f1 (Unity, 2022), combined with the Varjo XR Plugin (Varjo Developer, 2023). A custom script was used that allowed the experimenter to select the experiment conditions from within Unity.

**FIGURE 1:**
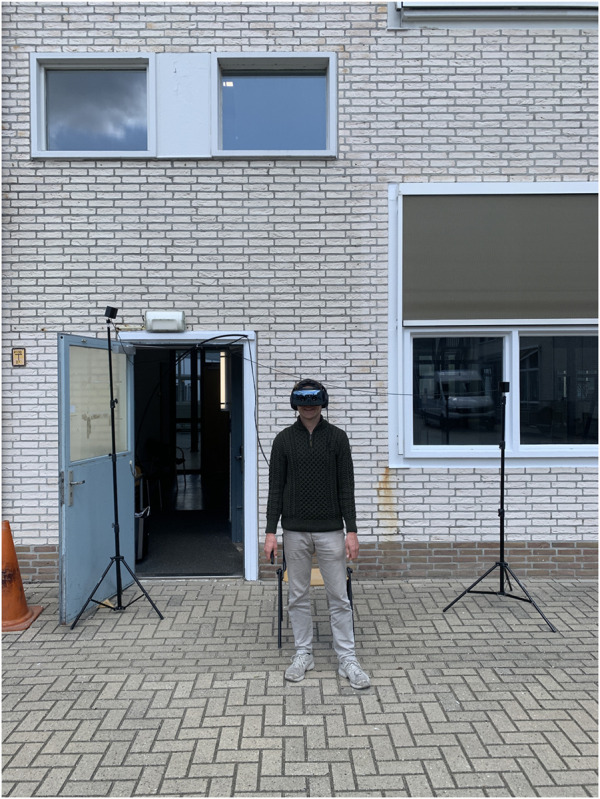
Participant wearing the tethered Varjo XR-3 headset.

AR was displayed by means of a Varjo XR-3 headset (Varjo, 2023). The Varjo XR-3 provides a 90 Hz refresh rate and a 115° horizontal field of view. The focus area, of 27° × 27°, was rendered at 70 pixels per degree on a µOLED display, providing 1920 × 1920 pixels per eye. The peripheral area was rendered at about 30 pixels per degree on an LCD, producing 2,880 × 2,720 pixels per eye. A pole was used to route the cables from the PC to the participant (Figure 1).

The Varjo XR-3 presented a virtual AV and virtual AR interfaces while depicting the real world by means of video pass-through. Within the headset configuration software ‘Varjo Base’, the imaging and exposure settings were set to automatic, and the highest image quality settings were selected. This proved to be feasible for our application without glitches or delays in data storage or visual animations.

Our initial intention was to anchor the virtual objects to the real world by means of object tracking and reference markers (Varjo Technologies, 2023). However, this approach proved to be unreliable in our outdoor setting, presumably due to the relatively empty environment and sunlight reflections on the markers. Instead, tracking for the headset was adopted, by means of two SteamVR Base Stations 2.0 (HTC VIVE, 2019), as depicted in Figure 1. Our solution was to position the *AR Origin* at a fixed position, based on the initial calibration position and orientation of the Varjo XR-3. Each time the hardware was set up, calibration of the HMD tracking was performed first. In Unity, an invisible *Ground place* was created, which functioned as a surface for the AV to drive on. The scale value of the AR Origin was set such that distances in the virtual world corresponded with distances in the real world. Unity was configured to allow participants to rotate their heads and look around, but not to translate or move through the environment. The height of the *Main Camera* above the ground plane was fixed at 1.70 m. Our outside-in tracking approach provides a simple and robust way to present virtual overlays onto the real world. However, the location of these overlays is not anchored to the real world, and can somewhat vary depending on the precise calibration of the setup.

A Logitech R400 wireless presenter was used as the remote control. The right arrow button was used by the participant as the button to indicate if the participant felt it was safe to cross. The receiver was connected via a USB port of the Alienware desktop.

The sound of the AV was transmitted through headphones that were plugged into the Varjo XR-3. The headphones did not include noise-cancelling, so surrounding real-world environment noise could still be heard.

### Participant information and task

Participants were provided with information about the procedure and tasks through a leaflet. They were informed that they would wear an AR headset, which uses cameras to capture the environment and can project virtual objects onto this real-world setting.

Participants were informed that they would stand at the side of a road and that their task was to press and hold the button when they felt it was safe to cross and to release it when they did not feel safe to cross. It was emphasised that participants should not physically cross the road. This method for measuring crossing intentions was previously introduced by De Clercq et al. (2019) and was considered favourable as it allows participants to respond almost immediately to changing conditions. In other research, we had participants actually cross (Tabone et al., 2023b) or communicate their crossing intention or ‘critical gap’ through hand gestures (Rodríguez Palmeiro et al., 2018) or by taking a single step (Epke et al., 2021). A drawback of these methods is the variability among participants in executing these actions, making it relatively challenging to extract their intentions or perceptions.

The leaflet also mentioned that, at the start of each trial, participants would see a circle (hereafter named ‘attention-attractor circle’) at one of three locations, i.e., either in front of them, to their left, or to their right. Participants were instructed to gaze at the circle for a duration of one second to initiate the trial. It was mentioned that a virtual vehicle would approach from the right, potentially stopping for the participant and possibly communicating this intent through various communication interfaces. Finally, it was mentioned that, after each trial, participants would be prompted with a question displayed in the AR environment, to which they were to provide a verbal response, and that this procedure would be repeated for a total of four different AR interfaces, plus a no-interface baseline condition. Participants were also informed that, following every block with a particular AR condition, the experimenter would ask open-ended questions about their experiences with the presented condition.

In previous research, the attention-attractor circles were used to impose visual distraction. This was done to examine whether different AR interfaces exhibit different robustness to distraction while the AR interface was active (Tabone et al., 2023b). Amongst others, Tabone et al. (2023b) found that the *Pedestrian lights HUD* supported crossing decisions even though the participants were visually distracted and had not glanced at the AV yet, because the HUD moved with the participant’s field of view. In the current study, the circles played a different role. In our case, the circles disappeared before the start of the trial, while the AR interface appeared 3.1 s later, which allowed participants to direct attention to the approaching AV before the activation of the AR interface. The purpose of the circles, in our case, was to reduce monotony of the task and to increase visual demands.

### Automated vehicle behaviour

In each trial, a virtual AV approached from the right, displaying one of two behaviours: non-yielding or yielding. The vehicle model asset had dimensions of 4.95 m in length, 2.10 m in width, and 1.35 m in height (Final Form Studio, 2021). The AV reflected light from a virtually positioned Sun, and the wheels rotated in accordance with the forward speed. Shadows were not simulated. Audio was incorporated to produce a speed-sensitive engine sound, with a Doppler effect applied.

When the trial began, the AV appeared approximately 45 m away from the participant, with the distance measured parallel to the road. Simultaneously, an attention-attractor stimulus in the form of a cyan circle ring appeared either in front of, to the left of, or to the right of the participant. After the participant looked at the circle for 1 s, the AV began driving at a speed of 30 km/h.

**FIGURE 2:**
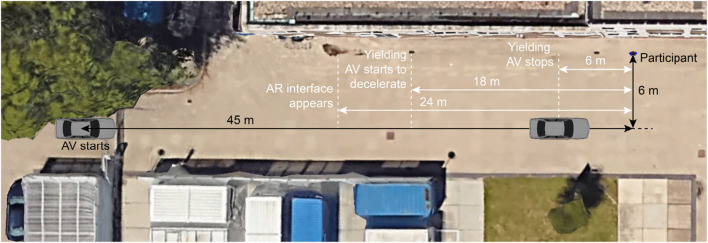
Top-down view of the experiment area (overlay drawn on an image from Google Earth, 2023).

Once 3.1 s had elapsed from the moment that the AV began driving, it passed an invisible trigger, prompting the AR interface to appear. In the yielding condition, the AV began decelerating 3.9 s from the start of its movement and came to a complete stop at an elapsed time of 7.8 s. The AV featured a slight forward pitch angle as it decelerated. In the non-yielding condition, the AV maintained a speed of 30 km/h and passed the participant 6.5 s from the beginning of its movement. Figure 2 provides an aerial view of the roadway, illustrating the sequence of events: the AV starting, the appearance of the AR interface, the point where the AV began decelerating, and where it came to a full stop.

**FIGURE 3:**
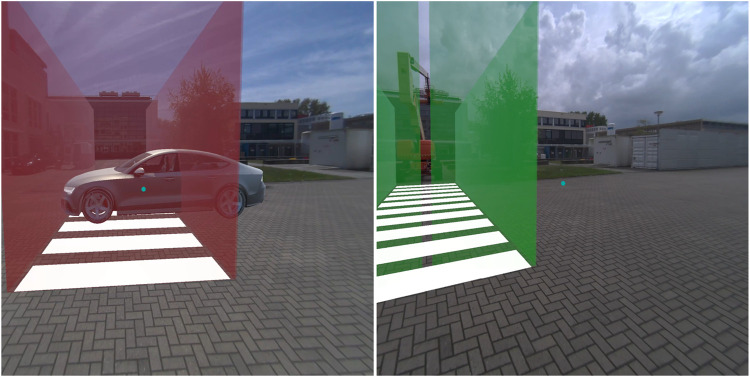
*Virtual fence* in the non-yielding (left) and yielding (right) condition.

**FIGURE 4:**
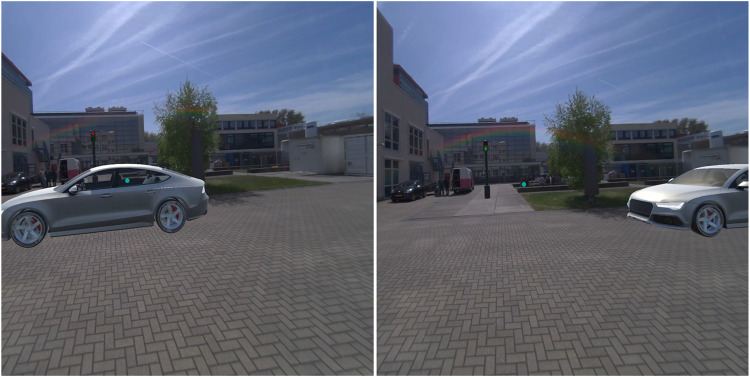
*Fixed pedestrian lights* in the non-yielding (left) and yielding (right) condition.

**FIGURE 5:**
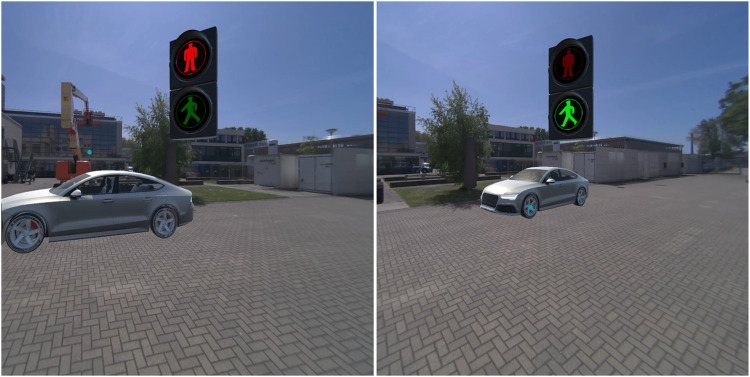
*Pedestrian lights HUD* in the non-yielding (left) and yielding (right) condition.

**FIGURE 6:**
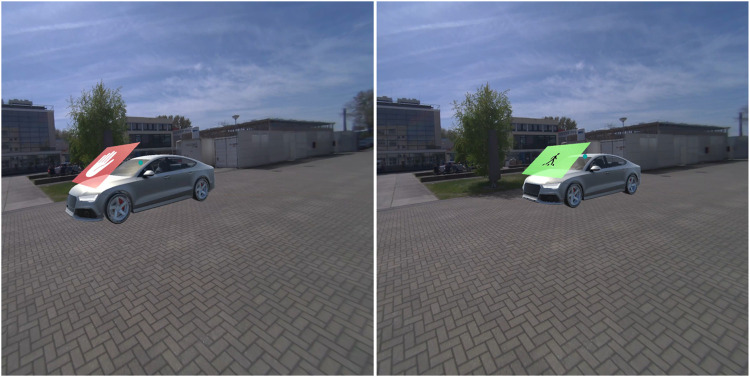
*Planes on vehicle* in the non-yielding (left) and yielding (right) condition.

### Augmented reality designs

The AR interfaces were adopted from earlier research in which these interfaces had been designed (Tabone et al., 2021b), and after minor modifications, presented to a large sample of online respondents (Tabone et al., 2023a), and subjected to experimental testing in an immersive virtual simulator (Tabone et al., 2023b). The nine AR interfaces were previously divided into three anchoring methods: world-locked, head-locked, and vehicle-locked (Tabone et al., 2023b). The present study focused on comparing four selected AR interfaces. Specifically, from each anchoring category, one was selected: *Virtual fence* (world-locked), *Pedestrian lights HUD* (head-locked), and *Planes on vehicle* (vehicle-locked). In addition, the *Fixed pedestrian lights* interface was selected. The reason for including this world-locked interface was to allow a comparison with the *Pedestrian lights HUD*. The four selected AR interfaces were as follows.

1. The *Virtual fence* consisted of a zebra crossing projected on the road combined with semi-translucent walls surrounding the zebra (Figure 3). The interface was 3.0 m tall, 2.5 m wide, and 7.5 m long. A semi-translucent gate positioned in front of the pedestrian opened in 1-s time after the interface appeared in the yielding condition, and remained closed in the non-yielding condition.
2. The *Fixed pedestrian lights* resembled existing traffic lights (Figure 4). It remained stationary across the street from where the pedestrian was positioned. The interface was 2.1 m tall and positioned 14 m from the pedestrian. It consisted of a pole with a box (0.20 m wide, 0.38 m tall) on top displaying either a lit-up red icon of a standing pedestrian, or a green icon of a walking pedestrian.
3. The *Pedestrian lights HUD* was similar to the *Fixed pedestrian lights* interface (Figure 5). However, instead of being world-locked, the same box was anchored to the user’s head. It was placed at 1 m distance from the participant’s head and off-centre by 30 cm upwards and to the right. The interface was rotated around the vertical axis in order to face the user.
4. The *Planes on vehicle* was a vehicle-mapped interface consisting of a red plane with a stop-hand icon or a green plane with an icon depicting a pedestrian crossing a zebra crosswalk (Figure 6). The 2.1 wide and 1.58 m tall plane hovered above the front of the AV so that the bottom of the plane aligned with the front bumper; it was tilted by 54° to be positioned parallel with the AV’s windshield.

In all four AR interfaces, colour was used to provide a redundant cue; for the non-yielding AVs this was pure red, and for yielding AVs this was pure green. The *Virtual fence* and *Planes on vehicle* were semi-transparent, to ensure that the AV or possibly other relevant objects remained visible to the participants.

### Experiment design

The experiment was of a within-subject design. Each participant was exposed to the four AR interfaces and a no-interface baseline condition, two AV behaviours (yielding and non-yielding), and three attention-attractor locations (left, middle, right).

Participants first completed two practice trials in the Baseline condition, one trial with a yielding AV one trial with a non-yielding AV. Each of the five interface conditions was presented in a separate block. Each block consisted of six trials: three with a yielding AV and three with a non-yielding AV. The three trials were conducted with the attention attractor at the left, right, or middle. This resulted in a total of 30 trials per participant (5 interface conditions × 2 yielding behaviours × 3 attention-attractor locations). The order of the five blocks, as well as the order of the six trials within each block, were counterbalanced using a Latin Square method.

The experiment lasted 45–60 min per participant, with the time spent wearing the headset amounting to approximately 30 min.

### Questionnaires and rating scales

After signing the consent form, participants completed an intake questionnaire, designed in Qualtrics (Qualtrics XM, 2023), to gather demographic information. The intake questionnaire also included items about affinity for technology (ATI scale, Franke et al., 2019), items about whether participants had experienced VR or AR headsets before, and a brief test for colour blindness (Ishihara, 1917). Some participants had completed the pre-experiment questionnaire before their scheduled experiment slot.

Next, participants were asked to read a leaflet with a short description of the experiment. A complementary oral explanation of the experiment was provided where needed. Subsequently, participants put on the Varjo XR-3 headset, were handed the remote button, and a multi-point eye-tracker calibration was conducted. After each trial, at exactly 10 s after the AV had started moving, a statement appeared in front of the participant: “*This interface/situation was intuitive for signalling: ‘Please do cross the road’.”* for the yielding condition, or “*This interface/situation was intuitive for signalling: ‘Please do not cross the road’.*” for the non-yielding condition. Participants verbally indicated to what extent they agreed on a scale from *Fully disagree* (1) to *Fully agree* (7).

After each block of six trials with a particular AR condition, a semi-structured interview was conducted regarding interface design qualities, the timing of the interface appearance, the preference between yielding and non-yielding state, and the participant’s wellbeing according to the misery scale (MISC; Bos, 2015).

After all five blocks, participants completed a post-experiment questionnaire in Qualtrics. This questionnaire contained items related to the AR experience and about AR interfaces in general. Participants were also asked to rank the five interface conditions in terms of their preference. Additionally, they were asked, for the four AR interfaces, to answer items regarding the intuitiveness of the green and red interfaces, convincingness of the green and red interface, interface trustworthiness, size (too small, too large), timing (too early, too late), clarity/understandability, and visual attractiveness, as well as a 9-item acceptance scale (Van der Laan et al., 1997), identical to Tabone et al. (2023a). The items used 7-point scales, except for the acceptance scale and adoption questions which use a 5-point semantic differential scale, and the ranking item. Open questions were also asked per interface condition to allow participants to justify their responses.

### Data recording and analysis

The data was stored at a frequency of 50 Hz. Firstly, we determined per trial what percentage of the time participants kept the response button pressed. This was done from the moment the AR interface appeared until the vehicle came to a stop (yielding AVs) or passed (non-yielding AVs).

Additionally, from the post-experiment questionnaire, we determined a composite score per AR interface, identical to how it was done by Tabone et al. (2023a). We calculated a composite score because the self-reports were strongly correlated, and we found no evidence of multiple underlying constructs (Tabone et al., 2023a). This composite score was calculated based on participants’ responses to 15 items, which included a 9-item acceptance scale and 6 additional items (1. Intuitiveness for non-yielding AVs, 2. Intuitiveness for yielding AVs, 3. Convincingness for non-yielding AVs, 4. Convincingness for yielding AVs, 5. Clarity/understandability, 6. Attractiveness). To calculate the composite score, we first concatenated the questionnaire results from the 28 participants across 4 AR conditions, resulting in a grand 112 × 15 matrix. We then standardised each of the 15 variables, so their mean became 0 and their standard deviation became 1. Next, we summed the standardised scores of the 15 items, resulting in a 112-element vector of total scores. These 112 scores were standardised again, providing a composite score for each participant and each AR interface. The preference rank, in which participants had to sort the five AR conditions from 1 (most preferred) to 5 (least preferred), was analysed as a separate item.

Finally, the post-trial intuitiveness ratings were averaged over 3 trials per participant so that for each AR interface and participant, and for both yielding and non-yielding AV, an intuitiveness score was available. Since the location of the attention-attractor circles did not appear to have a major influence on how the four AR interfaces were responded to, the results for the three trials per AR interface condition and yielding condition were averaged. In the statistical analyses, repeated-measures ANOVAs were used, with the AR condition as the independent variable. The findings are shown as means, complemented by 95% confidence intervals for within-subjects designs (Morey, 2008). For each of the nine measures, *post hoc* comparisons between conditions were conducted using paired-samples *t*-tests. An alpha value of 0.005 was chosen, which is more conservative than the usual 0.05, due to a maximum of 10 possible combinations of AR conditions that can be compared (4 + 3 + 2 + 1).

The interview results for each AR interface and participant were condensed into brief highlights by one of the authors (the experimenter). These highlights from all 28 participants were subsequently analysed and summarised per AR condition by another author using thematic analysis, with a focus on the clarity of the AR interface and participant satisfaction. The resulting summaries were checked with the interview transcripts (available for 16 out of the 28 participants) to ensure representativeness and were adjusted as needed. For each AR interface, one or two quotes (translated from Dutch) were selected for presentation alongside the summary.

## Results

The 28 participants performed a total of 840 trials. Four of 840 log files were unavailable or could not be used due to an experimenter error. For the post-experiment questionnaire, responses for the *Planes on vehicle* were unavailable for 1 of 28 participants.

Misery scores were low, with 26 of 28 participants reporting no symptoms (0 or 1 on the 11-point scale) and 2 of 28 participants reporting maximum scores of 3 and 4, respectively, at any time during the experiment. One of these two participants indicated feeling ill already before the experiment.

**FIGURE 7:**
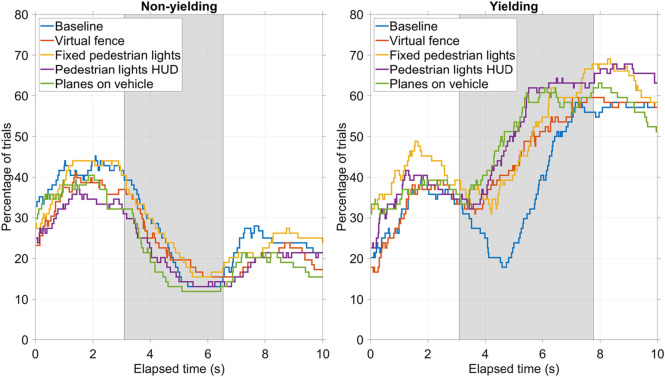
Button press percentage for the four AR interfaces and baseline condition, for non-yielding AVs (left) and yielding AVs (right). The grey background represents the interval between the moment the AR interface appeared (at 3.1 s) and the AV passed (non-yielding AVs, 6.5 s) or the AV came to a full stop (yielding AVs, 7.8 s).

Figure 7 shows the percentage of trials in which participants held down the response button. Interestingly, for both non-yielding AVs (Figure 7, left) and yielding AVs (Figure 7, right), a large portion of the participants (approximately 60%) did not hold down the response button at the beginning of the trial. The reasons for this are not clear, but it may be related to participants experiencing high workload or not being reminded to keep the button pressed at the start of the trial. A technological explanation, involving the wireless connection of the button device, cannot be ruled out either. Therefore, the results in Figure 7 should be considered in a relative context, comparing the five conditions with each other, rather than in absolute terms.

For the non-yielding AV (Figure 7, left), participants, on average, released the response button as the AV came closer, indicating that they felt less and less safe to cross. However, about 13% of the participants did not have the response button released while the vehicle passed.

For the yielding AVs (Figure 7, right), participants started pressing the response button after the AR interface appeared, at 3.1 s. For the baseline condition, this happened somewhat later, which can be explained by the fact that the AV only started to slow down at 3.9 s (thus, 0.8 s after the AR interfaces became visible). That is, in the baseline condition, participants could not anticipate that the AV was going to stop until it actually started to decelerate. From the button press percentages in the grey interval, it can be seen that the four AR interfaces were slightly more effective than the baseline condition.

**FIGURE 8:**
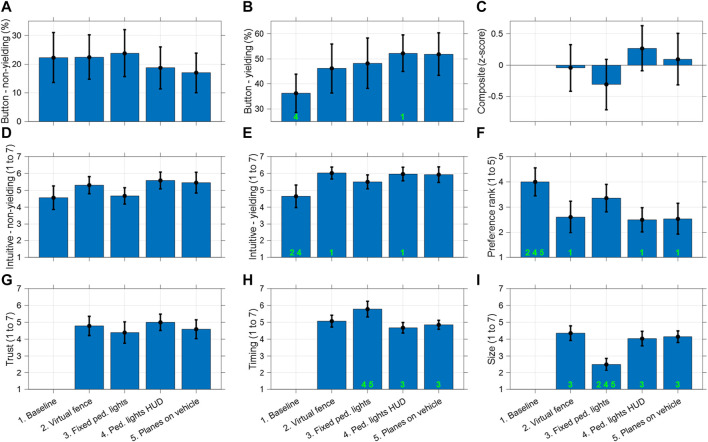
Means and 95% confidence intervals of participants’ **(A)** button press percentage for non-yielding AVs (%), **(B)** button press percentage for yielding AVs (%), **(C)** composite scores based on post-experiment self-reports (*z*-score), **(D)** post-trial intuitiveness scores for non-yielding AVs (1: fully disagree, 7: fully agree), **(E)** post-trial intuitiveness scores for yielding AVs (1: fully disagree, 7: fully agree), **(F)** preference rank (1: most preferred, 5: least preferred), **(G)** trust for decision-making (1: fully disagree, 7: fully agree), **(H)** interface trigger timing (1: too early, 7: too late), and **(I)** interface size (1: too small, 7: too large). The numbers in green indicate the conditions with which this condition shows a statistically significant difference (*p* < 0.005), according to paired-samples *t*-tests.

Figure 8 shows the button press percentages in the selected time interval for non-yielding AVs (A) and yielding AVs (B), as well as the mean composite scores calculated from 15 items of the post-experiment questionnaire (C), intuitiveness scores measured after each trial for non-yielding AVs (D) and yielding AVs (E), the preference rank (F), trust (G), timing (H), and size (I). The green numbers indicate pairs of conditions that exhibit statistically significant differences following a paired-samples *t*-test with Bonferroni correction. Full results from the paired-samples *t*-tests are accessible in the Data Repository mentioned in the Data Availability section.

The results presented in Figure 8 indicate that the baseline condition scored relatively poorly, as demonstrated by a low button press percentage for yielding AVs (B), low intuitiveness scores especially for non-yielding AVs (D) and yielding AVs (E), and on average the worst preference rank (F).

Among the four AR interfaces, there were no major differences, although the *Fixed pedestrian lights* scored relatively poorly. This is evident from the low composite score (C), low intuitiveness scores (D and E), and the poor preference rank (F) among the four AR interfaces. However, none of these effects were statistically significant. The largest effects between the four interface conditions were, however, observed regarding the perception of the physical variables timing and especially size. More specifically the *Fixed pedestrian lights* were perceived as clearly too small (I), and related to this, participants also believed that the traffic lights became visible too late (H); its timing was identical to that of the other three interfaces, but this assessment might be due to the fact that it took participants extra time to visually locate it, creating the impression that it became visible too late.

The results of the repeated-measures ANOVAs corresponding to the data presented in Figure 8 are shown in Table 1. The effect sizes (partial η^2^) were not particularly strong for the button presses (A and B), the composite score (C), or the trust score (G). However, they were fairly strong and statistically significant for the post-trial intuitiveness ratings (D and E), the preference rank (F), and as mentioned above, the timing (H) and size (I).

**TABLE 1:** Results of repeated-measures ANOVAs for the AR interface comparisons shown in Figure 8.

Dependent measure	Measurement moment	*df*	*F*	*p*	Partial η^2^
(A) Button press percentage, non-yielding AVs	During trials	4,108	0.57	0.684	0.02
(B) Button press percentage, yielding AVs	During trials	4,108	2.33	0.060	0.08
(C) Composite score	Post-experiment	3,78	1.65	0.185	0.06
(D) Intuitiveness, non-yielding AVs	Post-trial	4,108	2.88	0.026	0.10
(E) Intuitiveness, yielding AVs	Post trial	4,108	6.34	<0.001	0.19
(F) Preference rank	Post-experiment	4,108	5.72	<0.001	0.17
(G) Trust	Post-experiment	3,78	0.91	0.441	0.03
(H) Timing	Post-experiment	3,78	6.90	<0.001	0.21
(I) Size	Post-experiment	3,78	19.1	<0.001	0.42

Note. For (C), (G), (H), and (I), data for *Planes on vehicle* were missing for one participant.

The results of the post-block interviews are indicated below.

• *Baseline*. Without an interface, many participants felt the situation was more akin to real-life scenarios. They indicated that the absence of clear signals, or feelings of insecurity, without the interface caused them to take longer in making decisions. The factors influencing their decision-making were the AVs deceleration and speed. Additionally, the changing pitch of the AV’s sound during deceleration was reported to be a relevant cue.

“Well, at some point you see that car slowing down. Then you still remain a bit apprehensive, thinking, ‘Okay, it's really stopping.’ And only then do I decide to start walking.”

• *Virtual fence*. Participants mentioned that the size of the *Virtual Fence* made it stand out, and some even found it somewhat intimidating. The interface was generally perceived as clear, particularly the green signal. However, the red signal was confusing for some participants, who were unsure whether it warned them not to cross or indicated that the AV would stop. The zebra crossing within the interface also posed a dilemma: it seemed to encourage walking, but this was in conflict with the red walls. Additionally, some participants expressed concerns that the interface might obstruct the view of other potential road users.

“*Yes, so I found it a bit difficult at first, because you initially have those walls, which are red. So then you think, oh, is it red for them? And are you protected by those walls or something? And you're also in a zebra crossing. When I see a zebra crossing, I think, oh, I need to walk. So I found that a bit counterintuitive. But once you get used to it, I think you see it faster.*”

• *Fixed pedestrian lights*. The *Fixed pedestrian lights* were a familiar concept to participants. However, its appearance, timing, and placement of the traffic light posed challenges. Some participants reported they failed to notice it, especially during their first trial. Furthermore, its sudden appearance made it hard for participants to rely on. Additionally, a recurrent concern among participants was the need to switch gaze between the AV and the traffic light. Finally, participants suggested making the distinction between red and green clearer.

“*More unclear, because the pole only appears relatively late. So you’re really only focused on the car that’s approaching. And at first, I either saw the pole or I did not. You see the car approaching sooner, so then you start considering whether you’re going to cross or not. And only then do you see the pole.*”

• *Pedestrian lights HUD*. The *Pedestrian lights HUD* also provided a familiar interface. Its upper-right positioning in their field of view was reported to be both an advantage and a disadvantage. Specifically, some participants reported that the HUD required them to roll their eyes, which felt unnatural to them, while others appreciated the constant visibility and fixed position within their view. Its sudden appearance sometimes led to initial startles or distractions, and some participants commented that it was obstructive and blocked a portion of their view. Finally, as with the *Fixed pedestrian lights*, according to some participants, the distinction between the red and green signals (lit-up vs non-lit-up state) could have been clearer.

“Very clear, indeed. I'm not used to it, so that's why the first one might have been a bit unclear. Because I was thinking, should I now look or should I clearly know what the car is going to do? But after that, it's very clear, because it's just close by and you actually see it right away.”

• *Planes on vehicle*. Participants initially found the *Planes on vehicle* novel and stated it took some time to become accustomed to it. The green signal and icon were perceived as clear. However, there was some uncertainty regarding the red signal, with participants unsure whether the AV would stop. Many felt that the *Planes on vehicle* improved trust because the communication came directly from the AV. One colour blind person, however, did not immediately understand the meaning of this interface. Furthermore, some participants remarked that in hypothetical situations involving multiple vehicles, the effectiveness of the interface might decrease. It was suggested that, for optimal effectiveness in busier scenarios, all cars might need to adopt such an interface.

“*I do not know, it made me a bit nervous, because I thought okay. It seems very illogical that there's a very large sign in front of the car.*”

*“Better than the previous one. I liked that you only have to pay attention to one thing. So you just have to look at the car and it's clear whether you can cross or not. Instead of having to look somewhere else for a signal. It was just clearly on the car.”*

Finally, a recurring topic regarding the timing of the AR interfaces was that they were activated somewhat late (even though this was still 0.8 s before the vehicle began to brake). Participants suggested that the AR interfaces could appear earlier, possibly in a default state, so that they would know where to look in advance to make their crossing decision. Another theme that emerged from the interviews is that participants tended to verify the advice of the AR interfaces with the movement of the vehicle.

## Discussion

This study tested four AR interfaces for supporting the interaction with AVs among 28 human participants, complemented with a baseline condition without AR interface. For the research, we used actual AR in an outdoor environment, in contrast to previous research that used AR-in-VR. In our paradigm, participants stood outside and had to press a button as long as they felt safe to cross. The experiment was set up so that the AV was virtual; this way, we could offer the same vehicle movement and AR activation timings in every trial.

Some of the results did *not* match our previous studies. Specifically, a previous experiment in a CAVE environment (Tabone et al., 2023b) and a large-scale online survey (Tabone et al., 2023a) showed that among the AR interfaces tested, the world-locked *Fixed pedestrian lights* and head-locked *Pedestrian lights HUD* yielded relatively high intuitiveness ratings, while the vehicle-locked *Planes on vehicle* received lower ratings, though still positive. However, in the current study, the intuitiveness of the *Planes on vehicles* interface was rated highly, with average scores of 5.5 (*SD* = 1.6) for non-yielding AVs and 5.9 (*SD* = 1.2) for yielding AVs on a scale of 1–7, whereas the *Fixed pedestrian lights* interface received lower ratings, at 4.7 (*SD* = 1.7) for non-yielding AVs and 5.5 (*SD* = 1.2) for yielding AVs, respectively (see Figures 8D, E).

Although the relative ratings of the AR interfaces did not immediately correspond with prior research, the information-processing mechanisms did. For example, previous online research using animated video clips (Tabone et al., 2023a) and research using a virtual pedestrian simulator (Tabone and De Winter 2023) also found that a zebra crossing combined with the colour red can cause confusion, and that walls of the virtual fence may obstruct the view of other road users. Additionally, the fact that there can be an egocentric vs exocentric perspective confusion when a car emits a red signal is also known (Bazilinskyy et al., 2020), and the problem that world-locked interfaces, like the *Fixed pedestrian lights* in our case, cause divided attention, has also already been documented (e.g., Peereboom et al., 2023). The explanation for the relatively poor performance of the *Fixed pedestrian lights* in the present study seems to lie in more practical factors: We had placed it relatively far away from the participant, at 14 m. Furthermore, for most of the participants, an orange-coloured aerial work platform was present, located behind the Fixed pedestrian lights. This visual clutter could have further increased the difficulty in identifying the traffic light and discerning its status. This research thus shows that basic design decisions related to salience can have large effects on the effectiveness of AR interfaces.

The *Pedestrian lights HUD* also suffered from ‘practical limitations’; it was positioned slightly off-centre, leading to some discomfort as individuals had to adjust their gaze (see Plabst et al., 2022, for a similar phenomenon). Note that participants could only glance at the HUD if they rotated their eyes, as the HUD always followed the user’s head movement. The icons of the traffic light were not particularly salient in their illuminated ‘on’ state compared to their non-illuminated ‘off’ state, causing some participants to glance at the traffic light HUD instead of relying purely on their peripheral vision. Positioning the HUD towards a more central position would likely increase the risk of occlusion of relevant objects, which are typically centrally located in users’ fields of view. Participants experienced fairly low sickness scores, which differs from the higher scores observed in Peereboom et al.’s (2023) HUD study for pedestrians in VR. This difference may be attributed to our HUD being placed at a larger virtual distance (1.0 m, compared to their 0.36 m in Peereboom et al., 2023), reducing the accommodation-vergence conflict. However, it is anticipated that poorer task performance and/or nausea can occur in more dynamic situations, where the AR information is not locked to, or embedded in, the world. This concerns the use of HUDs while the user is walking through the environment, resulting in a moving background relative to static AR stimuli in the field of vision (Fukushima et al., 2020) or when standing still while the AR stimuli move in the field of view (Kaufeld et al., 2022). For future research, we suggest creating a HUD that does not require the user’s direct focus. For instance, the onset of a more bright coloured light, which can be unambiguously identified through peripheral vision, might offer a more comfortable experience for the user (e.g., Chaturvedi et al., 2019).

The *Fixed pedestrian lights* had the disadvantage that it required divided attention. This same disadvantage was mentioned in earlier experimental research in a CAVE environment, where participants took a relatively long time to visually identify the traffic lights (Tabone et al., 2023b). It might be the case that in the current real-world study, the task of identifying the traffic lights and the AV was even more challenging than in VR, because there is more visual clutter in the real world, such as other pedestrians and parked vehicles.

Indeed, we noticed that participants seemed to have difficulty with the experiment. For example, some participants had difficulty localising the attention-attractor circles or had to be reminded to look into these circles; participants also occasionally had to be reminded to press the response button if they felt safe to cross. One explanation for this forgetting is that we did not explicitly provide ‘press now’ instructions before each trial (e.g., Peereboom et al., 2023). Another explanation for the low press percentages is that the button, which operated through infrared light, might not have worked reliably in the outdoor environment. It was observed that when participants held the button in front of their body, instead of next to their body, missing values could result^1^. The button press percentages, as shown in Figure 7, resemble online crowdsourcing research on pedestrian-crossing decisions (e.g., Bazilinskyy et al., 2020; 2021; Sripada et al., 2021), where a portion of crowdworkers were apparently inattentive. Another possible explanation for the poor quality of button press data is that mental demands are higher with AR in the real world compared to AR-in-VR, because the real world is more cluttered, and the participant has more to keep track of, including perceiving real objects as well as maintaining postural stability and safety in the physical world. In turn, these observations suggest that simplicity and clarity of AR communication are likely even more important than in VR or online experiments.

A limitation of our study is that it used simulated AVs; in future traffic, actual AVs would need to communicate their stopping intentions wirelessly to the pedestrian’s AR headset. Furthermore, instead of outside-in tracking using base stations that emit infrared light, there would likely need to be a form of inside-out tracking, where the pedestrian’s headset detects objects in the environment (e.g., Bhakar et al., 2022). Although the Varjo XR-3 is a state-of-the-art AR device, there were some technical hiccups, such as a small number of occasions of loss of tracking, which caused VR objects to display an abrupt rotation, leading to some confusion or disorientation. Moreover, while the headset offered a large FOV, it was still more limited than natural vision without a headset. Finally, in the current study, only a single AV came from the right. Using multiple vehicles would increase realism, something that is especially relevant for vehicle-locked AR interfaces. On the other hand, the environment was realistic, with university employees and students walking around occasionally, as well as some maintenance vehicles being present. Adding false positives, like a green-coloured signal in combination with an AV that does not stop, could be useful to investigate (over-) reliance on the AR interfaces (see also Holländer et al., 2019; Kaleefathullah et al., 2022).

## Conclusion

In this study, different AR interfaces for AV-pedestrian interactions were assessed in an outdoor setting, distinguishing it from previous online and AR-in-VR research. The results showed that having an AR interface was generally preferred to no AR interface. Additionally, the results of post-trial interviews replicated previous information-processing-related findings, such as that a red-coloured surface can be confusing, since this cue can pertain to the AV or to the pedestrian. The experiment also showed that visual attention mechanisms are of key importance, with a world-locked traffic light causing challenges since pedestrians need to distribute attention between the approaching AV and the traffic light. Our findings also demonstrated the importance of practical design considerations, such as placement and salience, in determining the effectiveness of AR interfaces. Our results and observations further suggest that participants found the present task challenging, which, given the complex and cluttered nature of the real world as opposed to virtual environments, points to the need for simplicity and clarity in AR-based communication. Future research is thus advised to implement simple AR solutions, such as through a single coloured light visible in the periphery. Finally, it is recommended to explore forms of AR for pedestrians where the pedestrian can move around wirelessly. Such research could be a step towards AR for vulnerable road users, with real application.

## Data Availability

Raw data, MATLAB scripts used for the analyses, and material from the experiment are available at a public data repository https://doi.org/10.4121/a1f9f15c-1213-4657-8e4d-a154a725d747.

## Footnotes

1 Excluding trials where no button press was recorded is not advisable. This could introduce bias, as it is unclear whether participants genuinely did not feel safe crossing or if there was a technical issue.
